# Carotid Endarterectomy Versus Stenting for the Treatment of Patients With Carotid Artery Stenosis: An Updated Systematic Review and Meta-Analysis

**DOI:** 10.7759/cureus.35070

**Published:** 2023-02-16

**Authors:** Advait M Vasavada, Priyansha Singh, Arshia Firdaus, Dakshin Meenashi Sundaram, Malvik Patel, Ganeev Singh, Logesh Palanisamy, Sana Afreen Ansari, Sumaina Thummala, Harsh Pandya

**Affiliations:** 1 Internal Medicine, Shri M. P. Shah Government Medical College, Jamnagar, IND; 2 Internal Medicine, Smt. Nathiba Hargovandas Lakhmichand (NHL) Municipal Medical College, Ahmedabad, IND; 3 Surgery, Deccan College of Medical Sciences, Hyderabad, IND; 4 Internal Medicine, Employees' State Insurance Corporation (ESIC) Medical College & Post Graduate Institute of Medical Sciences and Research (PGIMSR), Chennai, IND; 5 Surgery, Government Medical College, Vadodara, IND; 6 Surgery, Sri Guru Ram Das Institute of Medical Sciences & Research, Amritsar, IND; 7 Internal Medicine, Government Mohan Kumaramangalam Medical College, Salem, IND; 8 Internal Medicine, Deccan College of Medical Sciences, Hyderabad, IND; 9 Internal Medicine, Karpaga Vinayaga Institute of Medical Sciences and Research Center, Maduranthakam, IND; 10 General Surgery, Shardaben General Hospital, Ahmedabad, IND

**Keywords:** stenting, carotid endarterectomy, revascularization, carotid artery stenosis, systematic review and meta analysis, post carotid endarterectomy, carotid stent

## Abstract

Carotid endarterectomy (CEA) is a surgical procedure that treats the narrowed carotid arteries, which may be narrowed by atherosclerosis. Stenting is the insertion of a wire mesh scaffold into the narrowed portion of the carotid artery to keep it open by preventing blood from clotting. Using the study done over 10 years back as a point of reference, this study will seek an update on an assessment comparing CEA and stenting in studies carried out between 2015 and to date. The PICOS (population, intervention, control, outcome, and study designs) criteria were used to construct a set of inclusion and exclusion guidelines. This meta-analysis and systematic review used two forms of investigative analysis; both quantitative and qualitative assessments. From the studies, stroke (95% CI: 0.51-0.71, P < 0.001), myocardial infarction (95% CI: 1.49-3.42, P = 0.001), and stroke or death analysis (95% CI: 0.53-0.77, P < 0.001) were noted to be significant. From the analysis, CEA was observed as having better treatment results in terms of stroke events and stroke or death incidences when compared to stenting. Carotid stenting was observed as having lower cases of myocardial infarctions when compared to endarterectomy.

## Introduction and background

Carotid endarterectomy (CEA) is a surgical procedure that treats the narrowed carotid arteries by the removal of parts of the artery or fatty plaques, which may be narrowed by atherosclerosis. It can be used in patients who have suffered from carotid artery stenosis, a condition that causes blood flow to slow down in the carotids. Stenting refers to percutaneous angioplasty, which is a treatment option in which a catheter is inserted into the blocked vessels and a meshed tube called a stent is placed [1]. CEA is considered for symptomatic patients with carotid stenosis who cannot undergo surgery [2].

Stenting is the insertion of a wire mesh scaffold into the narrowed portion of the carotid artery to keep it open by preventing blood from clotting [3]. Stents can be used alone or in combination with other interventions such as balloon angioplasty (balloon-like structure placed in narrowed portion), radiofrequency ablation (local heating), and peripheral artery bypass grafting (a surgical procedure where a leg vein or its part is replaced). A stent is placed within the artery to keep it open, thus decreasing the risk of a stroke [4]. The goal of this operation is to decrease blood flow through the narrowed portion of the artery, which reduces pressure and prevents a stroke. This procedure can be performed as an alternative to stenting in patients who do not have symptoms that indicate they need treatment with stents or angioplasty.

CEA and stenting for treating carotid artery stenosis are both widely used procedures [5]. However, there are significant differences between these two treatments. CEA is a surgical procedure that removes plaque from the carotid artery wall [1]. This procedure can be used as a stand-alone treatment for patients with severe stenosis or as a second-line treatment in cases where medical management has failed to improve symptoms or reduce stroke risk. Stenting involves the insertion of a dilator or stent into the arteries to open up blockages. This procedure may be performed alone or combined with CEA depending on individual patient circumstances and needs.

Studies have been done on this topic; among them is a study done by Moresoli et al. [6]. Moresoli et al. analyzed the results of stenting or endarterectomy for asymptomatic carotid artery stenosis in a systematic review and meta-analysis. They found that carotid stenting was associated with a higher rate of major adverse cardiovascular events (MACE) than endarterectomy. They also found no significant difference between the two procedures regarding death or stroke. However, they did find that patients undergoing stenting had less risk of death or stroke than those undergoing endarterectomy [6]. In another study by Guo et al., the authors compared the outcomes between redo stenting and endarterectomy for patients with in-stent stenosis after carotid artery stenting [7]. They found no significant difference between the two procedures regarding death or stroke. However, they did find that patients undergoing redo stenting had less risk of death or stroke than those undergoing endarterectomy. In past studies, both Moresoli et al. and Guo et al. conducted a meta-analysis of randomized controlled trials to compare the effectiveness of CEA with that of carotid stenting for asymptomatic carotid artery stenosis. The results were consistent in that both studies concluded that the two procedures are equally effective treatment options for patients with symptomatic carotid artery stenosis [6,7]. The first study was done on patients who had been referred to a hospital's vascular surgery center and were found to have asymptomatic carotid artery stenosis after undergoing a routine diagnostic ultrasound examination.

CEA and carotid stenting are viable options for patients with symptomatic or asymptomatic carotid artery stenosis. In a systematic review and meta-analysis of past studies, Texakalidis et al. found that endarterectomy was associated with better clinical outcomes than stenting [8]. Not only was there a greater reduction in the size of the artery after the endarterectomy (1 mm versus 0.6 mm), but there was also an improvement in symptoms such as dizziness and palpitations [8]. In contrast, Yuan et al. found that stenting was more effective than endarterectomy at reducing the size of the artery by 0.7 mm, but there were no differences between the two procedures in terms of symptom relief [9]. This suggests that although both procedures can lead to similar reductions in size, they also have other benefits that may make one procedure more preferable over another for certain patients [9].

## Review

Aims and objectives

Using the study by Meier et al. (2010) as a point of reference, this study will seek to get an update on an assessment comparing CEA and stenting in studies carried out between 2015 and to date (January 20, 2023) [10]. An analysis of the recent studies will be conducted individually, then a comparison will be conducted using a subgroup analysis with the current studies and those that were used in the study by Meier et al. [10].

Methods

Study Design

Preferred Reporting Items for Systematic Reviews and Meta-Analyses (PRISMA) guidelines were used in this meta-analysis and systematic review. The preparation for this systematic review also included the use of PRISMA extensions published in the Cochrane Handbook for Systematic Reviews and extensions [11].

Search Strategy

This study identified Cochrane Central, PubMed, and MEDLINE as the primary electronic databases for research. Supplementation of the available articles for the review was done using Google Scholar. The search strategy used keywords, keyword combinations, Medical Subject Heading (MeSH) terms, field tags, Boolean operators "AND" and "OR," and truncations. Search strings were built from these elements to ensure an accurate acquisition of the best articles. Table 1 illustrates the keywords and search strategies used in each database. Identified articles were sought to get the most relevant articles for this study.

**Table 1:** Databases and search strategy MeSH: Medical Subject Heading.

Databases	Keywords	Search strategy	Filters
PubMed	Carotid endarterectomy, carotid stenting, common carotid artery, carotid artery, constriction, treatment, carotid artery stenosis	(((("carotid endarterectomy"[All Fields] OR (("carotid artery, common"[MeSH Terms] OR ("carotid"[All Fields] AND "artery"[All Fields] AND "common"[All Fields]) OR "common carotid artery"[All Fields] OR ("carotid"[All Fields] AND "artery"[All Fields]) OR "carotid artery"[All Fields] OR "carotid arteries"[MeSH Terms] OR ("carotid"[All Fields] AND "arteries"[All Fields]) OR "carotid arteries"[All Fields] OR ("carotid"[All Fields] AND "artery"[All Fields])) AND "stenosis*"[All Fields])) AND "stenting*"[All Fields]) OR ("Endovascular"[All Fields] AND "stent*"[All Fields])) AND "patient*"[All Fields] AND "OR"[All Fields] AND ("endarterectomy"[MeSH Terms] OR "endarterectomy"[All Fields] OR "endarterectomies"[All Fields]) AND ("constriction, pathologic"[MeSH Terms] OR ("constriction"[All Fields] AND "pathologic"[All Fields]) OR "pathologic constriction"[All Fields] OR "stenosi"[All Fields] OR "stenosis"[All Fields]) AND ("carotid artery, common"[MeSH Terms] OR ("carotid"[All Fields] AND "artery"[All Fields] AND "common"[All Fields]) OR "common carotid artery"[All Fields] OR ("carotid"[All Fields] AND "artery"[All Fields]) OR "carotid artery"[All Fields] OR "carotid arteries"[MeSH Terms] OR ("carotid"[All Fields] AND "arteries"[All Fields]) OR "carotid arteries"[All Fields] OR ("carotid"[All Fields] AND "artery"[All Fields])) AND ("stent s"[All Fields] OR "stentings"[All Fields] OR "stents"[MeSH Terms] OR "stents"[All Fields] OR "stent"[All Fields] OR "stented"[All Fields] OR "stenting"[All Fields]) AND ("Endovascular"[All Fields] AND ("stent s"[All Fields] OR "stentings"[All Fields] OR "stents"[MeSH Terms] OR "stents"[All Fields] OR "stent"[All Fields] OR "stented"[All Fields] OR "stenting"[All Fields])) AND ("clinical trial"[Publication Type] OR "randomized controlled trial"[Publication Type])) AND (clinicaltrial[Filter] OR randomizedcontrolledtrial[Filter])	Free full text, clinical trials, human studies, English only, timeframe 2015-2023
MEDLINE	Carotid endarterectomy, carotid stenting, carotid artery stenosis	(Carotid endarterect*.tw AND carotid stent*.tw) AND "treatment" AND carotid artery steno*.tw	Free full text, clinical trials, human studies, English only, timeframe 2015-2023
PMC (PubMed Central)	Carotid endarterectomy, carotid stenting, treatment, carotid artery stenosis	(((("carotid endarterectomy"[All Fields] OR (("carotid artery, common"[MeSH Terms] OR ("carotid"[All Fields] AND "artery"[All Fields] AND "common"[All Fields]) OR "common carotid artery"[All Fields] OR ("carotid"[All Fields] AND "artery"[All Fields]) OR "carotid artery"[All Fields] OR "carotid arteries"[MeSH Terms] OR ("carotid"[All Fields] AND "arteries"[All Fields]) OR "carotid arteries"[All Fields] OR ("carotid"[All Fields] AND "artery"[All Fields])) AND "stenosis*"[All Fields])) AND "stenting*"[All Fields]) OR ("Endovascular"[All Fields] AND "stent*"[All Fields])) AND "patient*"[All Fields] AND "OR"[All Fields] AND ("endarterectomy"[MeSH Terms] OR "endarterectomy"[All Fields] OR "endarterectomies"[All Fields]) AND ("constriction, pathologic"[MeSH Terms] OR ("constriction"[All Fields] AND "pathologic"[All Fields]) OR "pathologic constriction"[All Fields] OR "stenosi"[All Fields] OR "stenosis"[All Fields]) AND ("carotid artery, common"[MeSH Terms] OR ("carotid"[All Fields] AND "artery"[All Fields] AND "common"[All Fields]) OR "common carotid artery"[All Fields] OR ("carotid"[All Fields] AND "artery"[All Fields]) OR "carotid artery"[All Fields] OR "carotid arteries"[MeSH Terms] OR ("carotid"[All Fields] AND "arteries"[All Fields]) OR "carotid arteries"[All Fields] OR ("carotid"[All Fields] AND "artery"[All Fields])) AND ("stent s"[All Fields] OR "stentings"[All Fields] OR "stents"[MeSH Terms] OR "stents"[All Fields] OR "stent"[All Fields] OR "stented"[All Fields] OR "stenting"[All Fields]) AND ("Endovascular"[All Fields] AND ("stent s"[All Fields] OR "stentings"[All Fields] OR "stents"[MeSH Terms] OR "stents"[All Fields] OR "stent"[All Fields] OR "stented"[All Fields] OR "stenting"[All Fields])) AND ("clinical trial"[Publication Type] OR "randomized controlled trial"[Publication Type])) AND (clinicaltrial[Filter] OR randomizedcontrolledtrial[Filter])	Open access, Free full text, clinical trials, human studies, English only, timeframe 2015-2023
Google Scholar	Carotid endarterectomy, carotid stenting, treatment, carotid artery stenosis	"Carotid endarterectomy" AND "carotid stenting" AND "treatment" OR "carotid artery stenosis"	Timeframe of 2015-2023

Eligibility Criteria

The researchers selected eligibility guidelines for the studies to be included in this systematic review. The PICOS (population, intervention, control, outcome, and study designs) criteria were used to construct a set of inclusion and exclusion guidelines. The population considered in the study was patients with carotid artery stenosis. The exposure for the study was the use of CEA, while the comparator for the study was carotid stenting. The studies considered were randomized control trials. Only English-published articles or those translated were considered for inclusion.

Data Extraction

Two researchers (Priyansha and Dakshin) conducted the extraction of data. A pre-designed Excel worksheet was used in the recording of extracted data. Information on the authors, year of publication, demographics, outcomes, and the results of the included studies was extracted. Engagement between the two researchers was constant to ensure the results' congruence. A third party (Arshia) quelled disputes that arose.

Statistical Analysis

This meta-analysis and systematic review used two forms of investigative analysis; both quantitative and qualitative assessments. Literal analysis was used to analyze the included studies' qualitative findings systematically. Review Manager 5.4 (RevMan 5.4) was used in the meta-analysis of the extracted data. The data analysis was used to assess the periprocedural death, myocardial infarctions, stroke, and post-procedural stroke. An assessment of two combinations was also conducted, which included death or stroke and death, stroke, or myocardial infarctions. A subgroup analysis was also conducted to get an update on the studies that were conducted after 2010. In addition, the analysis sought to find the odds ratio at a 95% confidence interval. T2, I2, and H2 statistics were used to determine heterogeneity among the included studies. Forest plots were used in the presentation of the outcomes of the study, while funnel plots were used to assess the symmetry of the distribution of the study. P-value indicated the test results' significance level, with P ≤ 0.05 indicating a significant difference. Random-effects model was used and our rationale to use it was not just based on heterogeneity but the overall criteria for choosing the model as illustrated by Tufanaru et al. [12]. The shortcomings of this approach are addressed in the limitation section.

Results

Study Selection

**Figure 1:**
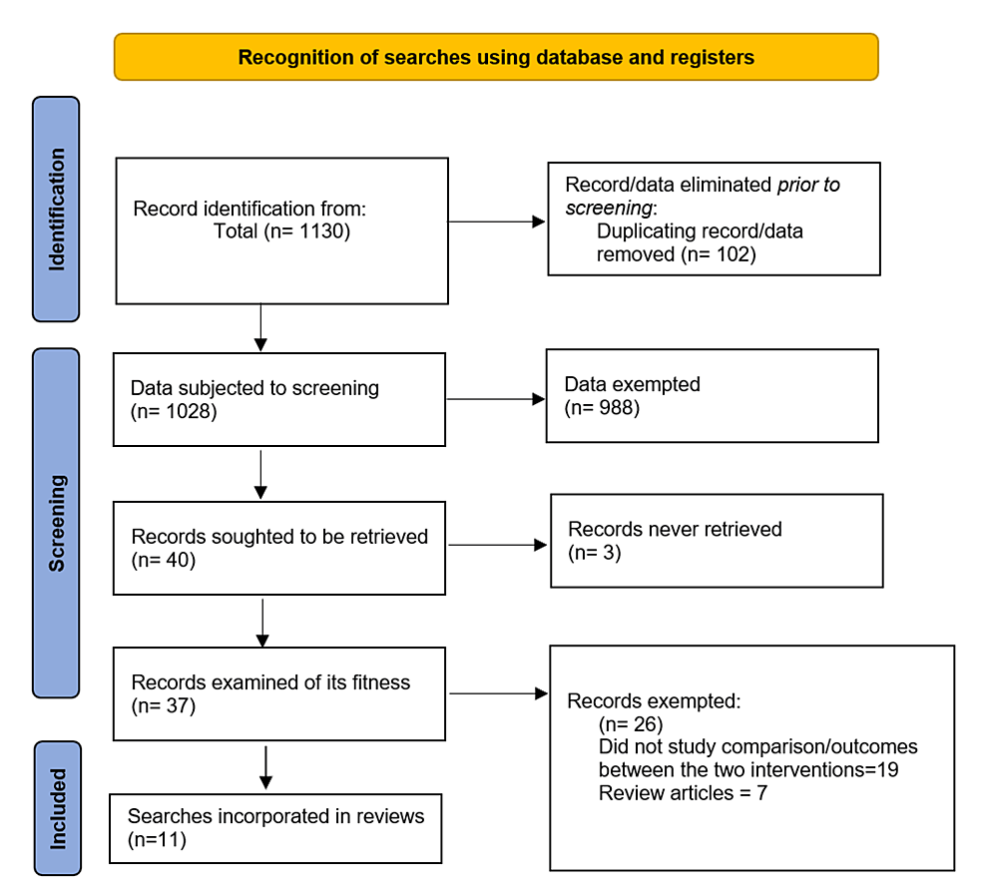
PRISMA flow diagram detailing the study selection process

From the electronic databases, 1130 studies were identified. From these, 102 studies were excluded as duplicates with 1028 studies remaining. The remaining studies were then screened using titles and abstracts to determine their suitability. From these, 988 studies were excluded, and 40 studies remained. Three studies were not retrieved due to the lack of access to the journal article. Further screening was conducted remaining with 37 studies. These studies were assessed for their suitability, which exempted 26 articles. Eleven studies were selected for use in the meta-analysis and systematic review. Figure 1 below shows the selection process.

Study Characteristics

Study characteristics identified from the selected studies were filled in a predesigned Excel sheet (Microsoft Corporation, Redmond, WA) and displayed in Table 2 below.

**Table 2:** Study characteristics Studies [13-23]. QVSS: Questionnaire for Verifying Stroke-Free Status.

Author	Year	Study design	Demographic	Endarterectomy	Stenting	Results
Reiff et al. [13]	2021	Randomized, controlled, open, multicenter trial	Age: 70 (64-75) vs. 70 (63-75); male: 151 vs. 143; coronary disease: 70 vs. 72	n = 203	n = 197	Stroke: 8 vs. 8; periprocedural stroke: 4 vs. 3; after 30 days: 3 vs. 3
Meschia et al. [14]	2022	Multicenter, randomized carotid revascularization endarterectomy vs. stenting trial	Age: 69.8 +/- 8 vs. 69.5 +/- 7.9; female: 32.8% vs. 37.4%	n = 406	n = 420	Adjudicated stroke: 44; QVSS symptoms: 183; adjudicated stroke or symptoms: 199
Halliday et al. [15]	2021	International, multicenter, randomized trial	Male: 1273 vs. 1272; age < 70: 893 vs. 909, > 70: 921 vs. 902	n = 1814	n=1811	Stroke: 41 vs. 61; myocardial infarctions: 12 vs. 5; death, stroke, or myocardial infarction: 55 vs. 67; death: 2 vs. 2; death or any stroke: 47 vs. 63
Yang et al. [16]	2021	Prospective, multicenter cohort study	Age: 64.2 vs. 65.5; male: 359 vs. 575, North China: 390 vs. 490	n = 418	n = 656	Death, stroke, or myocardial infarctions: 21 vs. 25; stroke: 17 vs. 21; death: 2 vs. 0; myocardial infarctions: 4 vs. 4
Matsumura et al. [17]	2022	Randomized controlled trial	Age: 68.25 vs. 67.7; >65 years: 643 vs. 1135; male: 574 vs. 1021; White: 845 vs. 1501	n = 907	n = 1637	Periprocedural: stroke, myocardial infarction, death: 29/891 vs. 52/1620; death: 2/891 vs. 2/1620; stroke: 13/891 vs. 43/1620; myocardial infarction: 15/891 vs. 9/1620; death or stroke: 14/891 vs. 44/1620
Rosenfield et al. [18]	2016	Prospective multicenter trial	Age: 67.9 vs. 67.7; >65 years: 261 vs. 764; male: 207 vs. 666, White: 327 vs. 985	n = 364	n =1089	Myocardial infarction, death, stroke: 9/348 vs. 35/1072; death: 1/348 vs. 1/1072; stroke: 5/348 vs. 30/1072; myocardial infarction: 3/348 vs. 5/1072; composite complications: 17/364 vs. 31/1089; death, stroke: 6/348 vs. 31/1072
Brott et al. [19]	2016	Randomized controlled trial	Age: 69.0; male: 65.2%; White: 93.2%; asymptomatic: 47.2%	n = 1240	n = 1262	Myocardial infarction, death, stroke: 56 vs. 66; myocardial infarction: 28 vs. 14; stroke: 29 vs. 52; after periprocedural period myocardial infarction, death, stroke: 41 vs. 42; stroke: 29 vs. 52; death, stroke: 29 vs. 55
Featherstone et al. [20]	2016	International, multicenter, randomized controlled, open, prospective clinical trial	Age:70 vs. 70; male: 606 vs. 601	n = 857	n = 853	Stroke, death, myocardial infarction: 44 vs. 72; stroke: 35 vs. 65; death: 7 vs. 19; death, stroke: 36 vs. 68
Bonati et al. [21]	2018	Parallel-group randomized trial	Age:70.6 vs. 70.0; men: 561 vs. 513; women: 232 vs. 224,	n = 793	n = 737	Stroke: 22/723 vs. 39/735
Bonati et al. [22]	2015	International, multicenter, randomized clinical trial	Age: 70 vs. 70; male: 606 vs. 601	n = 857	n = 853	Periprocedural death, stroke: 49 vs. 59; stroke: 72 vs. 119; death: 129 vs. 153; post-procedural death, stroke: 27/811 vs. 24/752
Mannheim et al. [23]	2017	Randomized controlled trial	Age: 68 vs. 69; male: 48 vs. 45; smokers: 20 vs. 15	n = 68	n = 68	Periprocedural: death: 0 vs. 0; stroke: 1 vs. 2; infection: 1 vs. 0; cranial nerve injury: 1 vs. 0; long term: death: 4/67 vs. 4/65; stroke: 0/67 vs. 0/65

Statistical Analysis

**Figure 2:**
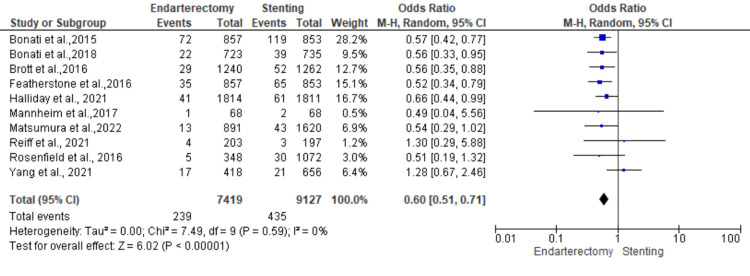
Stroke analysis forest plot Studies [13,15-23].

**Figure 3:**
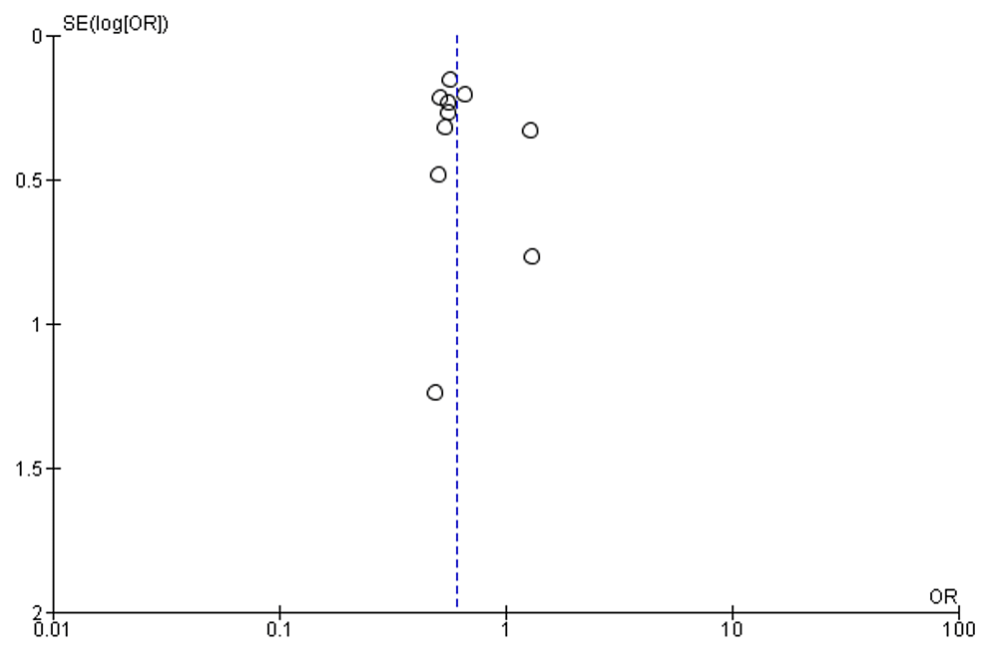
Stroke analysis funnel plot Studies [13,15-23].

Stroke: Ten studies were used in this outcome analysis [13,15-23]. A total of 16,546 patients were included in the analysis on stroke, randomized as 7419 patients in endarterectomy treatment and 9127 in the stenting treatment. The odds ratio was 0.60 (0.51-0.71) at a 95% confidence interval. The test for overall effect indicates Z = 6.02 (P < 0.001), which demonstrates a significant difference between the two treatment modalities. In addition, the test has a heterogeneity of df = 9 (P = 0.59) and I2 = 0%. Figures 2, 3 below are the forest and funnel plots from the meta-analysis.

**Figure 4:**
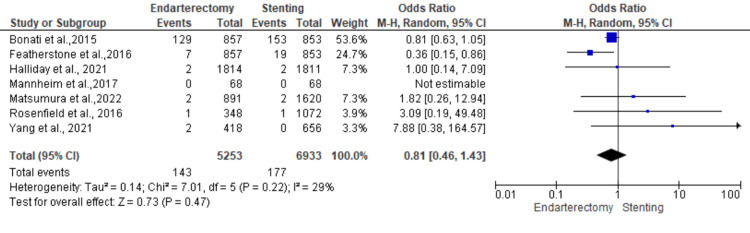
Death analysis forest plot Studies [15-18,20,22,23].

**Figure 5:**
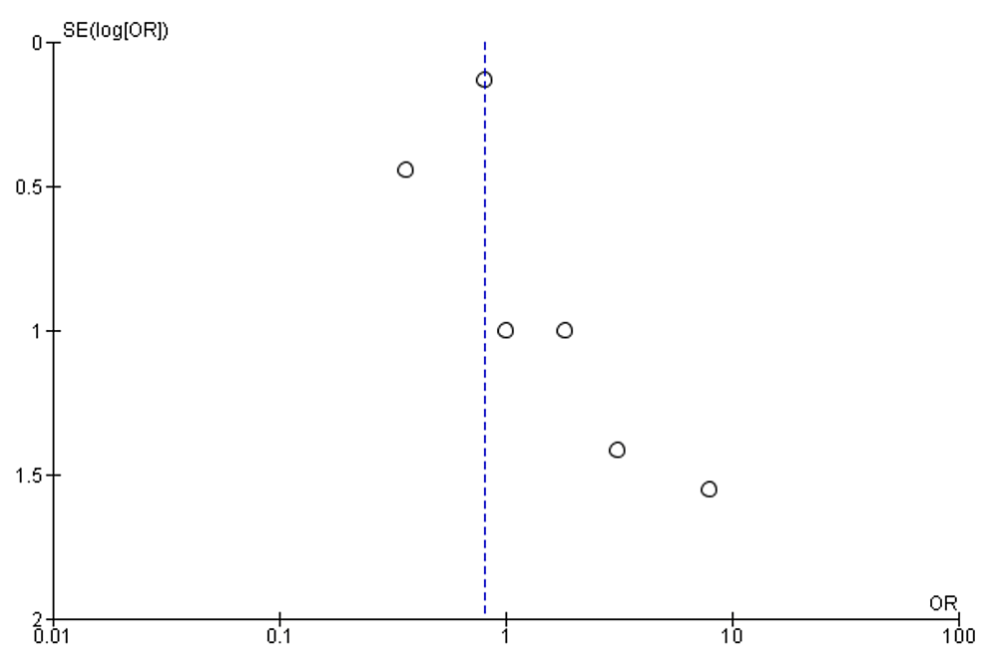
Death analysis funnel plot Studies [15-18,20,22,23].

Death: Seven studies were used in this outcome analysis [15-18,20,22,23]. A total of 12,186 patients were included in the analysis on death, randomized as 5253 patients in endarterectomy treatment and 6933 in the stenting treatment. The odds ratio was 0.81 (0.46-1.43) at a 95% confidence interval. The test for overall effect indicates Z = 0.73 (P = 0.47), which does not demonstrate any significant difference between the two treatment modalities. In addition, the test has a heterogeneity of df = 5 (P = 0.22) and I2 = 29%. Figures 4, 5 below are the forest and funnel plots from the meta-analysis.

**Figure 6:**
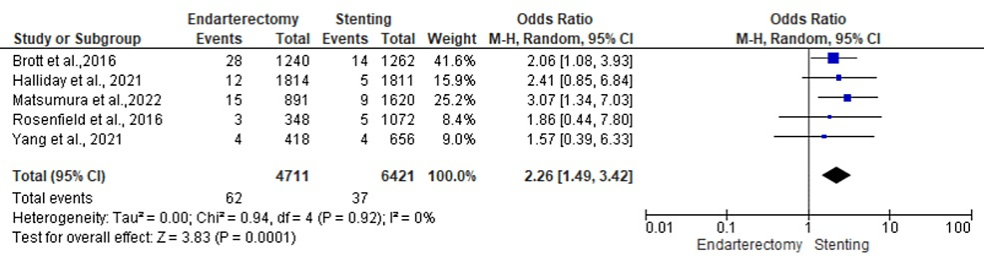
Myocardial infarction analysis forest plot Studies [15-19].

**Figure 7:**
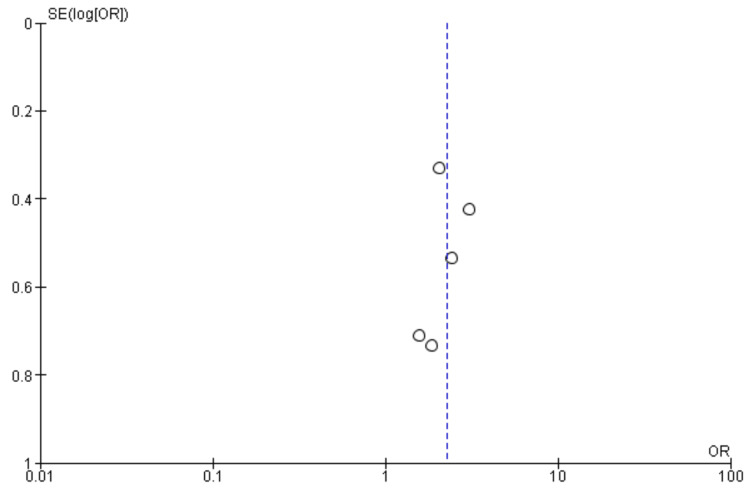
Myocardial infarction analysis funnel plot Studies [15-19].

Myocardial infarction: Five studies were used in this outcome analysis [15-19]. A total of 11,132 patients were included in the analysis on myocardial infarctions, randomized as 4711 patients in endarterectomy treatment and 6421 in the stenting treatment. The odds ratio was 2.26 (1.49-3.42) at a 95% confidence interval. The test for overall effect indicates Z = 3.83 (P = 0.001), which demonstrates a significant difference between the two treatment modalities. In addition, the test has a heterogeneity of df = 4 (P = 0.92) and I2 = 0%. Figures 6, 7 below are the forest and funnel plots from the meta-analysis.

**Figure 8:**
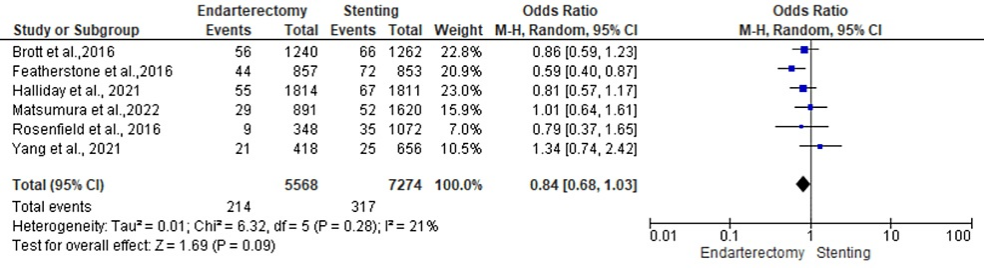
Stroke, death, or myocardial infarction analysis forest plot Studies [15-20].

**Figure 9:**
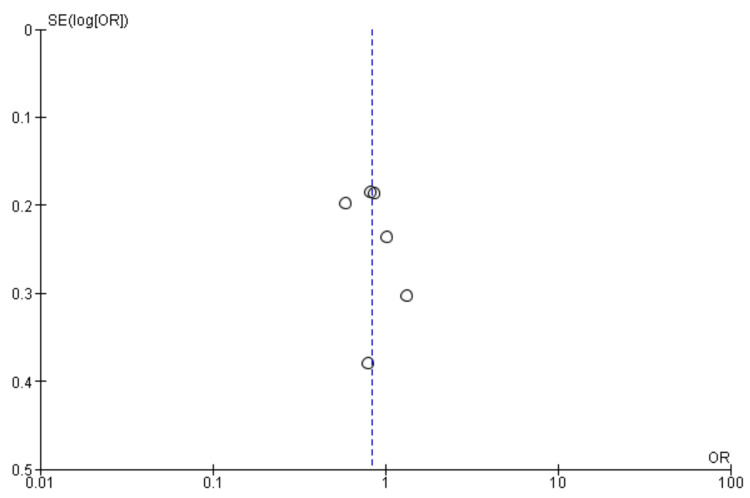
Stroke, death, or myocardial infarction analysis funnel plot Studies [15-20].

Stroke, death, or myocardial infarction: Six studies were used in this outcome analysis [15-20]. A total of 12,842 patients were included in the analysis on stroke, death, or myocardial infarction, randomized as 5568 patients in endarterectomy treatment and 7274 in the stenting treatment. The odds ratio was 0.84 (0.68, 1.03) at a 95% confidence interval. The test for overall effect indicates Z = 1.69 (P = 0.09), which does not demonstrate any significant difference between the two treatment modalities. In addition, the test has a heterogeneity of df = 5 (P = 0.28) and I2 = 21%. Figures 8, 9 below are the forest and funnel plots from the meta-analysis.

**Figure 10:**
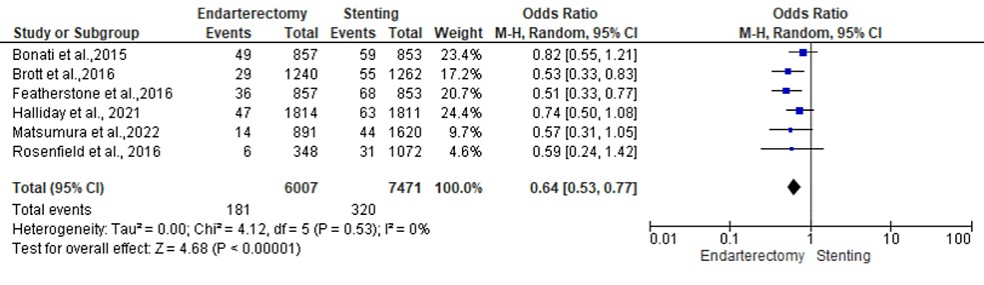
Stroke or death analysis forest plot Studies [15,17-20,22].

**Figure 11:**
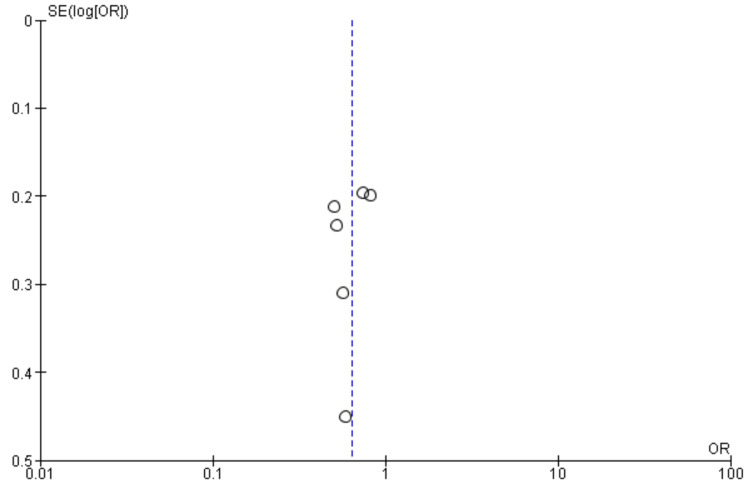
Stroke or death analysis funnel plot Studies [15,17-20,22].

Stroke or death: Six studies were used in this outcome analysis [15,17-20,22]. A total of 13,478 patients were included in the analysis on stroke or death, randomized as 6007 patients in endarterectomy treatment and 7471 in the stenting treatment. The odds ratio was 0.64 (0.53-0.77) at a 95% confidence interval. The test for overall effect indicates Z = 4.68 (P < 0.001), which demonstrates a significant difference between the two treatment modalities. In addition, the test has a heterogeneity of df = 5 (P = 0.53) and I2 = 0%. Figures 10, 11 below are the forest and funnel plots from the meta-analysis.

**Figure 12:**
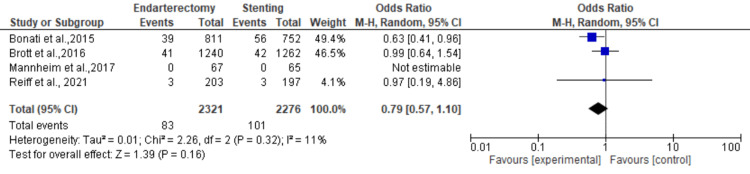
Post-procedural stroke analysis forest plot Studies [13,19,22,23].

**Figure 13:**
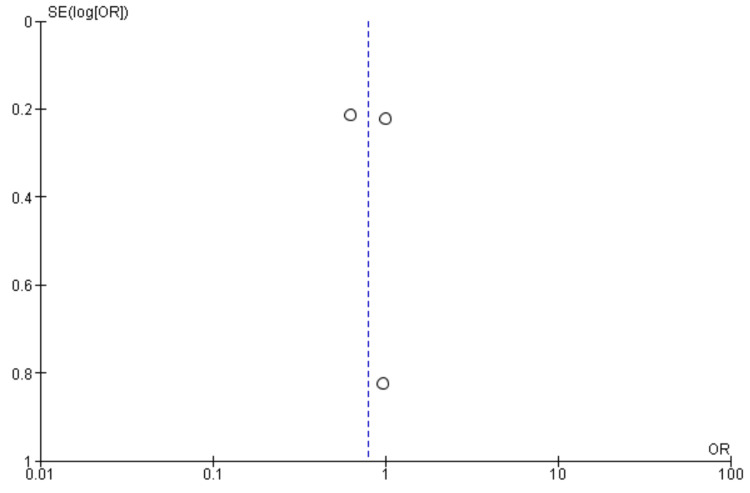
Post-procedural stroke analysis funnel plot Studies [13,19,22,23].

Post-procedural stroke: Four studies were used in this outcome analysis [13,19,22,23]. A total of 4597 patients were included in the analysis on post-procedural stroke, randomized as 2321 patients in endarterectomy treatment and 2276 in the stenting treatment. The odds ratio was 0.79 (0.57-1.10) at a 95% confidence interval. The test for overall effect indicates Z = 1.39 (P = 0.16), which does not demonstrate any significant difference between the two treatment modalities. In addition, the test has a heterogeneity of df = 2 (P = 0.32) and I2 = 11%. Figures 12, 13 below are the forest and funnel plots from the meta-analysis.

Subgroup Analysis

A subgroup analysis was conducted as a means to compare results acquired in the study by Meier et al. in 2010. This analysis was conducted as a means to get an update on current studies. The analysis was conducted from 10 studies that were analyzed in the study by Meier et al. (2010) [10,24-33].

**Figure 14:**
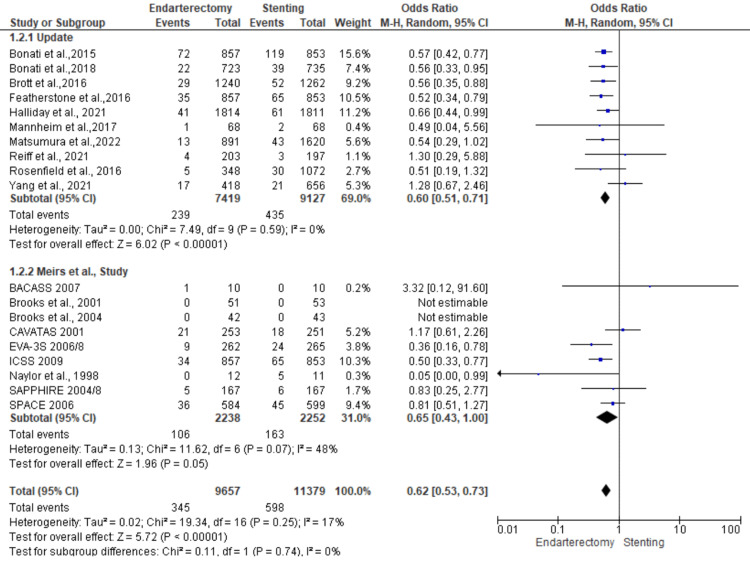
Stroke subgroup analysis forest plot Studies [10,13,15-33].

**Figure 15:**
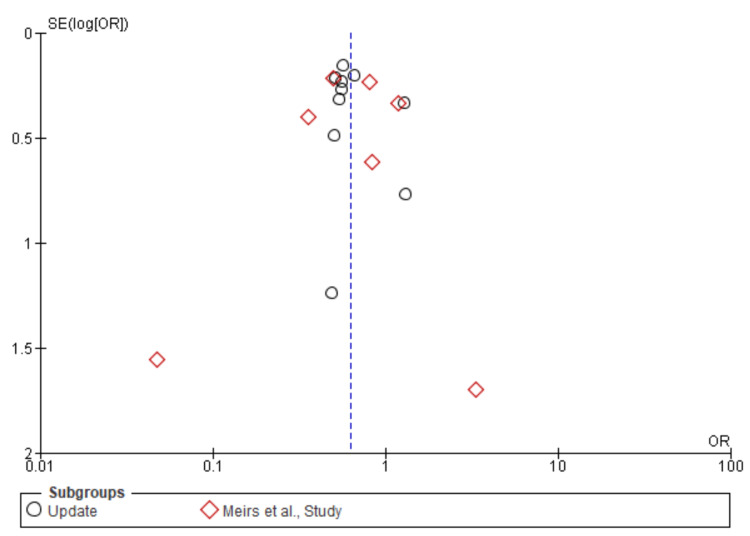
Stroke subgroup analysis funnel plot Studies [10,13,15-33].

Stroke: A total of 21,036 patients were included in the subgroup analysis on stroke, randomized as 9657 patients in endarterectomy treatment and 11,379 in the stenting treatment. The odds ratio was 0.62 (0.53-0.73) at a 95% confidence interval. The test for overall effect indicates Z = 5.72 (P < 0.001), which demonstrates a significant difference between the two treatment modalities. In addition, the test has a heterogeneity of df = 16 (P = 0.25) and I2 = 17%. The test for subgroup differences was df = 1 (P = 0.74) and I2 = 0%. Figures 14, 15 below are the forest and funnel plots from the meta-analysis.

**Figure 16:**
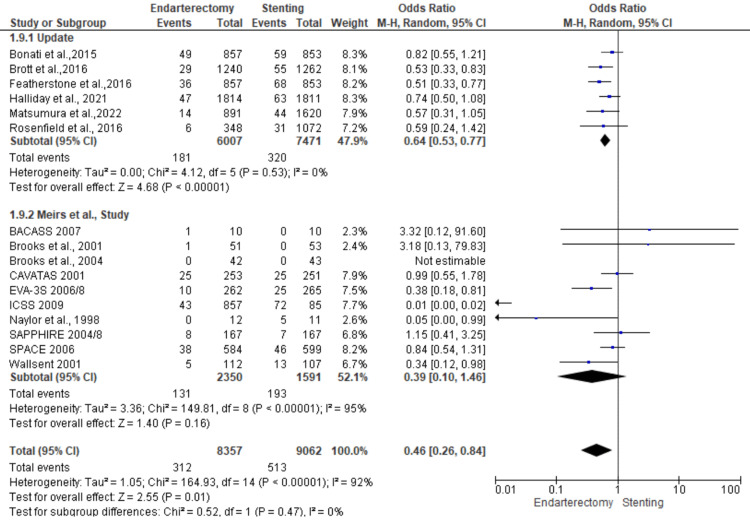
Death or stroke subgroup analysis forest plot Studies [10,13,15-33].

**Figure 17:**
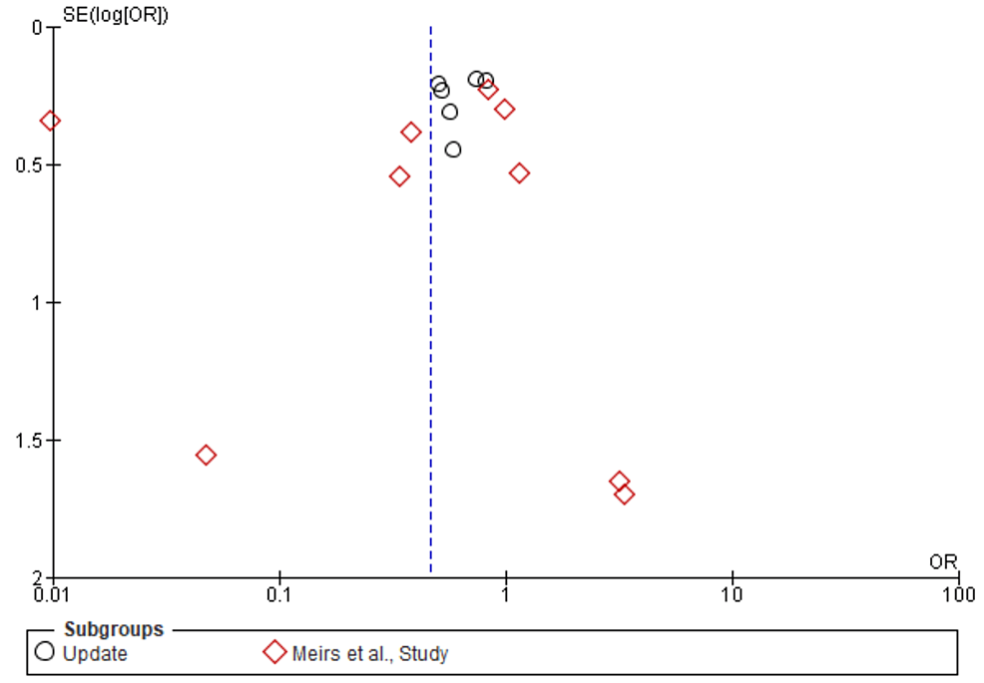
Death or stroke subgroup analysis funnel plot Studies [10,13,15-33].

Death or stroke: A total of 17,419 patients were included in the subgroup analysis on death or stroke, randomized as 8357 patients in endarterectomy treatment and 9062 in the stenting treatment. The odds ratio was 0.46 (0.26-0.84) at a 95% confidence interval. The test for overall effect indicates Z = 2.55 (P = 0.01), which demonstrates a significant difference between the two treatment modalities. In addition, the test has a heterogeneity of df = 14 (P < 0.001) and I2 = 92%. The test for subgroup differences was df = 1 (P = 0.47) and I2 = 0%. Figures 16, 17 below are the forest and funnel plots from the meta-analysis.

**Figure 18:**
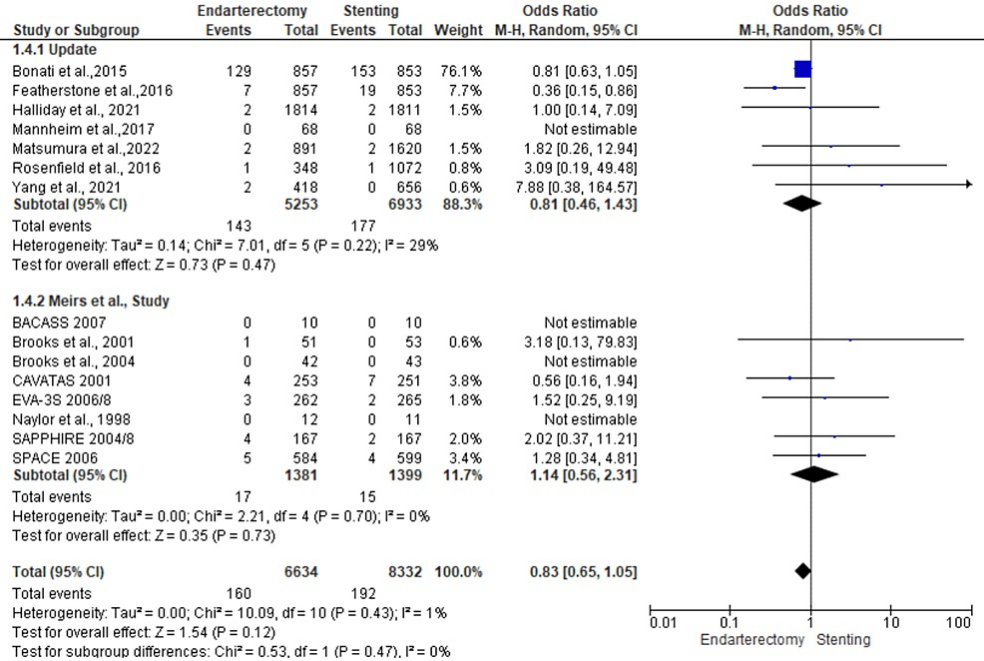
Death subgroup analysis forest plot Studies [10,13,15-33].

**Figure 19:**
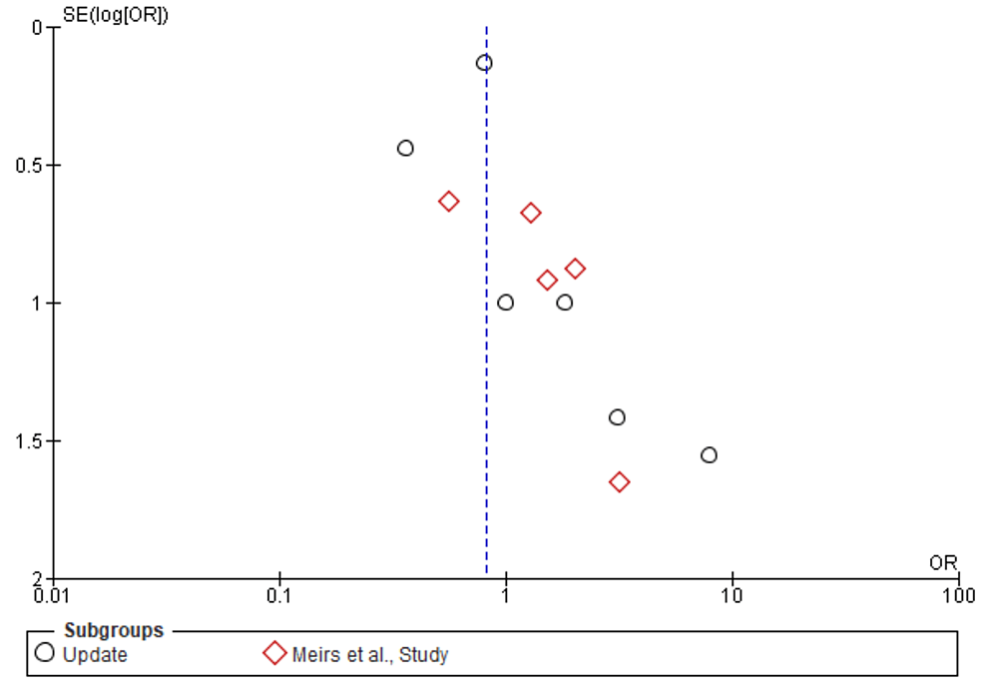
Death subgroup analysis funnel plot Studies [10,13,15-33].

Death: A total of 14,966 patients were included in the subgroup analysis on death, randomized as 6634 patients in endarterectomy treatment and 8332 in the stenting treatment. The odds ratio was 0.83 (0.65-1.05) at a 95% confidence interval. The test for overall effect indicates Z = 1.54 (P = 0.12), which does not demonstrate any significant difference between the two treatment modalities. In addition, the test has a heterogeneity of df = 10 (P = 0.43) and I2 = 1%. The test for subgroup differences was df = 1 (P = 0.47) and I2 = 0%. Figures 18, 19 below are the forest and funnel plots from the meta-analysis.

**Figure 20:**
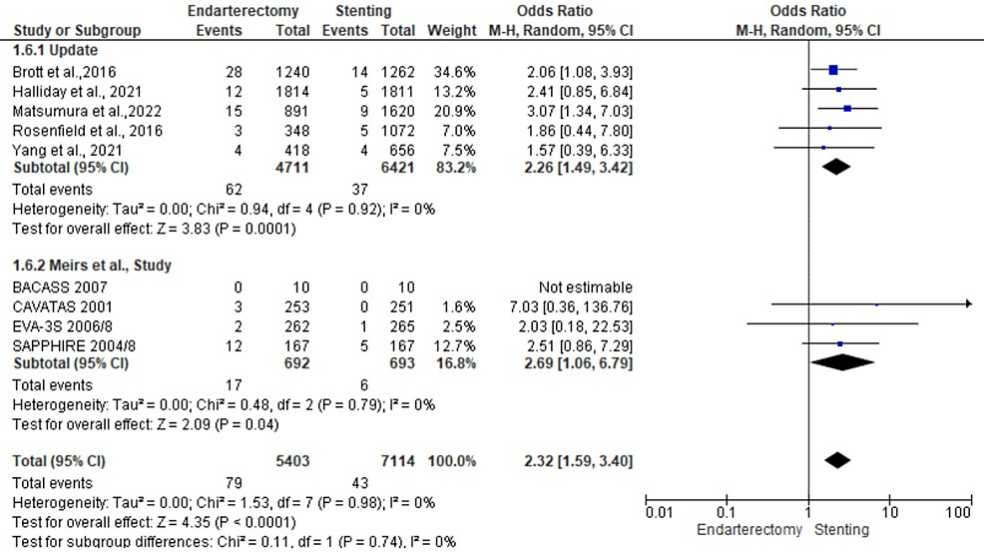
Myocardial infarction subgroup analysis forest plot Studies [10,13,15-33].

**Figure 21:**
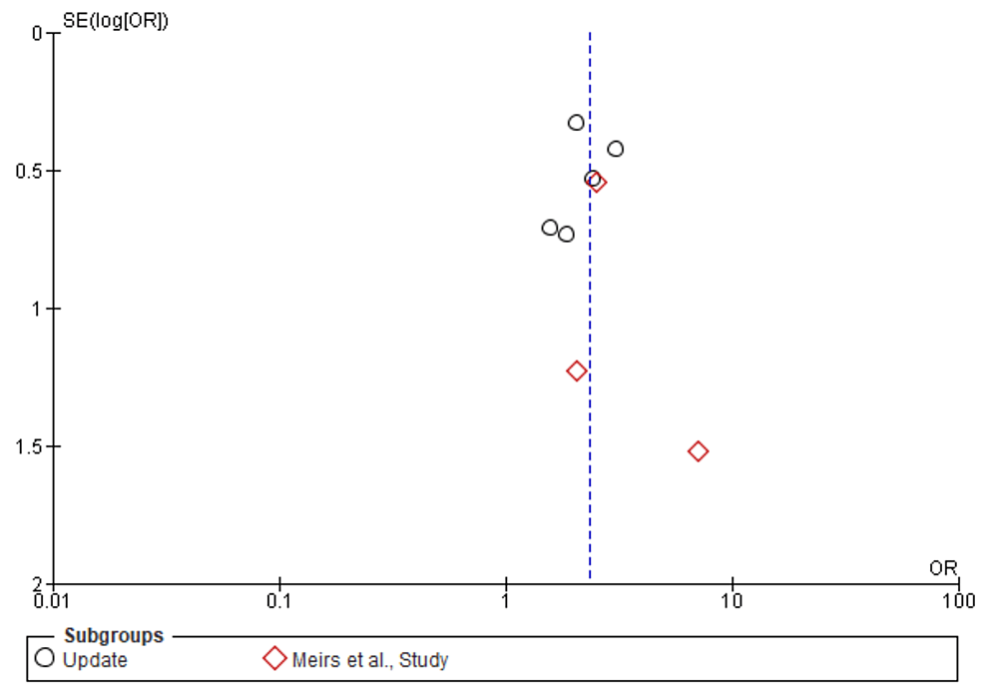
Myocardial infarction subgroup analysis funnel plot Studies [10,13,15-33].

Myocardial infarction: A total of 12,517 patients were included in the subgroup analysis on stroke, randomized as 5403 patients in endarterectomy treatment and 7114 in the stenting treatment. The odds ratio was 2.32 (1.59-3.40) at a 95% confidence interval. The test for overall effect indicates Z = 4.35 (P < 0.001), which demonstrates a significant difference between the two treatment modalities. In addition, the test has a heterogeneity of df = 7 (P = 0.98) and I2 = 0%. The test for subgroup differences was df = 1 (P = 0.74) and I2 = 0%. Figures 20, 21 below are the forest and funnel plots from the meta-analysis.

Discussion

This study sought to get an update on the study by Meier et al. (2010) on the comparison between CEA and stenting in the treatment of carotid artery stenosis [10]. In the study, an analysis is conducted on the effects of the treatments on stroke, mortality, myocardial infarction, and their combinations at a periprocedural period, which was considered as the period during which the treatment was used and within 30 days. A post-procedural assessment of analysis was conducted on stroke alone as data on the other study points were not available. The post-procedural period was 30 days after the initial use of the treatment. A subgroup analysis was conducted to identify any progression or differences between the treatments as seen in the study by Meier et al. (2010) and the current studies from 2015 to January 2023.

From the studies, stroke (95% CI: 0.51-0.71, P < 0.001), myocardial infarction (95% CI: 1.49-3.42, P = 0.001), and stroke or death analysis (95% CI: 0.53-0.77, P < 0.001) were noted to be significant. From the analysis, CEA was observed as having better treatment results in terms of stroke events and stroke or death incidences when compared to stenting. Carotid stenting was observed as having lower cases of myocardial infarctions when compared to endarterectomy. Death (95% CI: 0.46-1.43, P = 0.47), stroke, death, or myocardial infarctions (95% CI: 0.68-1.03, P = 0.09), and post-procedural stroke (95% CI: 0.57-1.10, P = 0.16) were reported to have no significant differences between the two modalities.

From the subgroup analysis conducted, it was reported that a significant difference was reported in the analysis of stroke (95% CI: 0.53-0.73, P < 0.001), death or stroke (95% CI: 0.26-0.84, P = 0.01), and myocardial infarctions (95% CI: 1.59-3.40, P < 0.001). Similar to the current studies, death (95% CI: 0.65-1.05, P = 0.12) had no significant difference between the two modalities. The analysis of the study from Meier et al. (2010) and current studies had similarities in all areas. CEA was observed as having better results in stroke and stroke or death analysis. Stenting on the other hand was reported as having better results in terms of myocardial infarctions.

Reiff et al. (2021) note that the risk of patients getting a stroke was correlated to having severe stenosis of the carotid artery as well as having an occlusion [13]. Cerebrovascular events in the study were reported as being affected by the degree of contralateral stenosis. The risk was seemingly increased with the use of interventional treatment. Just as reported from our analysis, the study observed that the risk of stroke within the first 30 days was significantly higher when carotid stenting was used. Halliday et al. (2021) report that the treatment intervention used in their study was limited to patients who needed it [15]. Similar to our analysis, the study reported that CEA and stenting had no difference in their effects on death. Stroke occurrence was reported to be higher whenever stenting treatment was used as compared to endarterectomy. Myocardial infarctions from studies analyzed were reported to occur at a higher rate in endarterectomy treatment as compared to stenting.

Limitations

The article is limited due to constrictions in using non-blinded studies only as blinded studies based on surgical procedures are unethical. The statistical analysis was conducted largely based on a random-effects model, which might be a source of errors and inaccuracies in the analysis. In the case of heterogeneity, some experts may argue fixed effects model would be a more suitable model for such a study. But our rationale to use the random-effects model was not just based on heterogeneity (the reason reviewers suggest we use fixed-effects) but the overall criteria tested and supported by the literature published in the *International Journal of Evidence-Based Healthcare* [12]. If our study goal was to not generalize or compare outcomes, we might have used a fixed-effects model. But our study compares past studies and the results were meant to ensure validity outside of the study for which the fixed-effects model is not a good fit. Also, the number of studies included in our review is more than five. In summary, basing the decision of which model to use just on the basis of heterogeneity is not the best idea in our opinion. We have added a detailed explanation of why random effects were used in our revision and we will highlight and explain the issues raised here in the limitation section so that the readers are well-informed regarding the thought process of the authors and the rationale of our analysis. Moreover, there is a chance of unreliable results due to the varying small populations utilized in individual studies in addition to different treatment modalities used. Also, the time range of follow-up in the studies is not long term; hence, it is vital that future studies take into consideration the relationship of time with the outcomes of the interventions if longer durations are considered. Very few studies focused on the predictors and patient characteristics that could also benefit patient selection for the appropriate modality [34].

## Conclusions

As observed from our study, endarterectomy was noted to have fewer harmful effects when compared to stenting. This was observed in the analysis of stroke and death or stroke analysis. Stenting was noted to have better outcomes in terms of myocardial infarctions. The subgroup analysis helped identify the similarities and differences between our current study and prior studies. Ultimately, the study observed that both treatment methods increased risk effects to an extent for the patients and more evidence in the form of long-term outcomes research would be suitable to determine the best modality of treatment. Furthermore, predictors and patient characteristics of adverse outcomes would benefit the selection of the treatment modality.

## References

[1] Rerkasem A, Orrapin S, Howard DP, Rerkasem K (2020). Carotid endarterectomy for symptomatic carotid stenosis. Cochrane Database Syst Rev.

[2] Uno M, Takai H, Yagi K, Matsubara S (2020). Surgical technique for carotid endarterectomy: current methods and problems. Neurol Med Chir (Tokyo).

[3] Lamanna A, Maingard J, Barras CD (2019). Carotid artery stenting: current state of evidence and future directions. Acta Neurol Scand.

[4] Avgerinos ED, Saadeddin Z, Humar R (2019). Outcomes of left renal vein stenting in patients with nutcracker syndrome. J Vasc Surg Venous Lymphat Disord.

[5] Müller MD, Lyrer P, Brown MM, Bonati LH (2020). Carotid artery stenting versus endarterectomy for treatment of carotid artery stenosis. Cochrane Database Syst Rev.

[6] Moresoli P, Habib B, Reynier P, Secrest MH, Eisenberg MJ, Filion KB (2017). Carotid stenting versus endarterectomy for asymptomatic carotid artery stenosis: a systematic review and meta-analysis. Stroke.

[7] Guo Z, Liu C, Huang K (2021). Meta-analysis of redo stenting versus endarterectomy for in-stent stenosis after carotid artery stenting. J Vasc Surg.

[8] Texakalidis P, Giannopoulos S, Jonnalagadda AK (2018). Carotid artery endarterectomy versus carotid artery stenting for restenosis after carotid artery endarterectomy: a systematic review and meta-analysis. World Neurosurg.

[9] Yuan G, Zhou S, Wu W, Zhang Y, Lei J, Huang B (2018). Carotid artery stenting versus carotid endarterectomy for treatment of asymptomatic carotid artery stenosis. Int Heart J.

[10] Meier P, Knapp G, Tamhane U, Chaturvedi S, Gurm HS (2010). Short term and intermediate term comparison of endarterectomy versus stenting for carotid artery stenosis: systematic review and meta-analysis of randomised controlled clinical trials. BMJ.

[11] Page MJ, McKenzie JE, Bossuyt PM (2021). The PRISMA 2020 statement: an updated guideline for reporting systematic reviews. Syst Rev.

[12] Tufanaru C, Munn Z, Stephenson M, Aromataris E (2015). Fixed or random effects meta-analysis? Common methodological issues in systematic reviews of effectiveness. Int J Evid Based Healthc.

[13] Reiff T, Eckstein HH, Mansmann U (2021). Contralateral stenosis and echolucent plaque morphology are associated with elevated stroke risk in patients treated with asymptomatic carotid artery stenosis within a controlled clinical trial (SPACE-2). J Stroke Cerebrovasc Dis.

[14] Meschia JF, Brott TG, Voeks J, Howard VJ, Howard G (2022). Stroke symptoms as a surrogate in stroke primary prevention trials: the CREST experience. Neurology.

[15] Halliday A, Bulbulia R, Bonati LH, Chester J, Cradduck-Bamford A, Peto R, Pan H (2021). Second asymptomatic carotid surgery trial (ACST-2): a randomised comparison of carotid artery stenting versus carotid endarterectomy. Lancet.

[16] Yang B, Ma Y, Wang T (2021). Carotid endarterectomy and stenting in a Chinese population: safety outcome of the revascularization of extracranial carotid artery stenosis trial. Transl Stroke Res.

[17] Matsumura JS, Hanlon BM, Rosenfield K, Voeks JH, Howard G, Roubin GS, Brott TG (2022). Treatment of carotid stenosis in asymptomatic, nonoctogenarian, standard risk patients with stenting versus endarterectomy trials. J Vasc Surg.

[18] Rosenfield K, Matsumura JS, Chaturvedi S (2016). Randomized trial of stent versus surgery for asymptomatic carotid stenosis. N Engl J Med.

[19] Brott TG, Howard G, Roubin GS (2016). Long-term results of stenting versus endarterectomy for carotid-artery stenosis. N Engl J Med.

[20] Featherstone RL, Dobson J, Ederle J (2016). Carotid artery stenting compared with endarterectomy in patients with symptomatic carotid stenosis (International Carotid Stenting Study): a randomised controlled trial with cost-effectiveness analysis. Health Technol Assess.

[21] Bonati LH, Gregson J, Dobson J (2018). Restenosis and risk of stroke after stenting or endarterectomy for symptomatic carotid stenosis in the International Carotid Stenting Study (ICSS): secondary analysis of a randomised trial. Lancet Neurol.

[22] Bonati LH, Dobson J, Featherstone RL (2015). Long-term outcomes after stenting versus endarterectomy for treatment of symptomatic carotid stenosis: the International Carotid Stenting Study (ICSS) randomised trial. Lancet.

[23] Mannheim D, Karmeli R (2017). A prospective randomized trial comparing endarterectomy to stenting in severe asymptomatic carotid stenosis. J Cardiovasc Surg (Torino).

[24] Hoffmann A, Engelter S, Taschner C (2008). Carotid artery stenting versus carotid endarterectomy - a prospective randomised controlled single-centre trial with long-term follow-up (BACASS). Schweiz Arch Neurol Psychiatr.

[25] Brooks WH, McClure RR, Jones MR, Coleman TC, Breathitt L (2001). Carotid angioplasty and stenting versus carotid endarterectomy: randomized trial in a community hospital. J Am Coll Cardiol.

[26] Brooks WH, McClure RR, Jones MR, Coleman TL, Breathitt L (2004). Carotid angioplasty and stenting versus carotid endarterectomy for treatment of asymptomatic carotid stenosis: a randomized trial in a community hospital. Neurosurgery.

[27] CAVATAS Investigators (2001). Endovascular versus surgical treatment in patients with carotid stenosis in the Carotid and Vertebral Artery Transluminal Angioplasty Study (CAVATAS): a randomised trial. Lancet.

[28] Mas JL, Trinquart L, Leys D (2008). Endarterectomy versus angioplasty in patients with symptomatic severe carotid stenosis (EVA-3S) trial: results up to 4 years from a randomised, multicentre trial. Lancet Neurol.

[29] Brown MM, Ederle J, Bonati LH, Featherstone RL, Dobson J (2009). Safety results of the International Carotid Stenting Study (ICSS): early outcome of patients randomised between carotid stenting and endarterectomy for symptomatic carotid stenosis. European Stroke Conference.

[30] Naylor AR, Bolia A, Abbott RJ (1998). Randomized study of carotid angioplasty and stenting versus carotid endarterectomy: a stopped trial. J Vasc Surg.

[31] Gurm HS, Yadav JS, Fayad P (2008). Long-term results of carotid stenting versus endarterectomy in high-risk patients. N Engl J Med.

[32] The SPACE Collaborative Group (2006). 30 day results from the SPACE trial of stent-protected angioplasty versus carotid endarterectomy in symptomatic patients: a randomised non-inferiority trial. Lancet.

[33] Alberts MJ (2001). Results of a multicenter prospective randomized trial of carotid artery stenting vs. carotid endarterectomy. Stroke.

[34] Nies KP, Smits LJ, Kassem M, Nederkoorn PJ, van Oostenbrugge RJ, Kooi ME (2021). Emerging role of carotid MRI for personalized ischemic stroke risk prediction in patients with carotid artery stenosis. Front Neurol.

